# A Bio-Realistic Finite Element Model to Evaluate the Effect of Masticatory Loadings on Mouse Mandible-Related Tissues

**DOI:** 10.3389/fphys.2017.00273

**Published:** 2017-05-09

**Authors:** Alexander Tsouknidas, Lucia Jimenez-Rojo, Evangelos Karatsis, Nikolaos Michailidis, Thimios A. Mitsiadis

**Affiliations:** ^1^Laboratory of Mechanical Engineering Systems, Department of Mechanical Engineering, University of Western MacedoniaKozani, Greece; ^2^Physical Metallurgy Laboratory, Department of Mechanical Engineering, Aristotle University of ThessalonikiThessaloniki, Greece; ^3^Orofacial Development and Regeneration, Institute of Oral Biology, ZZM, University of ZurichZurich, Switzerland; ^4^BETA CAE Systems S.A.Thessaloniki, Greece

**Keywords:** rodents, mandible, tooth, molar, incisor, craniofacial evolution, biomechanics, modeling

## Abstract

Mice are arguably the dominant model organisms for studies investigating the effect of genetic traits on the pathways to mammalian skull and teeth development, thus being integral in exploring craniofacial and dental evolution. The aim of this study is to analyse the functional significance of masticatory loads on the mouse mandible and identify critical stress accumulations that could trigger phenotypic and/or growth alterations in mandible-related structures. To achieve this, a 3D model of mouse skulls was reconstructed based on Micro Computed Tomography measurements. Upon segmenting the main hard tissue components of the mandible such as incisors, molars and alveolar bone, boundary conditions were assigned on the basis of the masticatory muscle architecture. The model was subjected to four loading scenarios simulating different feeding ecologies according to the hard or soft type of food and chewing or gnawing biting movement. Chewing and gnawing resulted in varying loading patterns, with biting type exerting a dominant effect on the stress variations experienced by the mandible and loading intensity correlating linearly to the stress increase. The simulation provided refined insight on the mechanobiology of the mouse mandible, indicating that food consistency could influence micro evolutionary divergence patterns in mandible shape of rodents.

## Introduction

The developmental pathways related to rodent skull morphogenesis have been extensively studied in the past years, identifying the effect of all traits related to their DNA sequence on the shape of the various skull bones, including mandible (Yamada et al., 1995; Colvin et al., 1996; Settle et al., 2003). Recent studies suggested that the phenotypic plasticity of epigenetic processes such as dietary aspects and muscle driven remodeling could favor the selection of pre-existing variances (Mavropoulos et al., 2004; Renaud et al., 2010). If this hypothesis holds true, then this micro-evolutionary pathway would be by far more direct in shape remodeling than the genotypic influences recruited in macro-evolutionary trends (Schluter, 1996). Although these morphological modifications to environmental influences (e.g., feeding ecology) can be detected by inbreeding laboratory strains, the trigger of this evolution is difficult to isolate since postnatal skull growth in rodents occurs preferentially in some parts of the mandible such as the mental foramen.

In order to consolidate this hypothesis, we undertook an *in-situ* approach that explores the importance of stress fields induced on the mouse mandible by masticatory loading. The exerted forces are expected to vary depending on the food stiffness and biting type (i.e., chewing or gnawing). Consequently, food consistency could result in evolutionary divergence patterns triggered through altered mandibular growth. However, the mandible of rodents is exposed to diurnal forces of high complexity and therefore the effects of mechanical loadings remain unclear due to limitations inherent to current experimental models. Computational methods may offer a good alternative to heuristic/experimental methodologies having the potential to answer important endured questions and confirm or not generalized assumptions on the mechanobiology of mandibles.

Over the past years Finite Element (FE) modeling has naturally evolved from traditional engineering disciplines to the study of living tissues, rapidly covering a broad spectrum of clinical applications (Tsouknidas et al., 2013, 2015). The wide acceptance of medical modeling by the academic community has enabled FE to rise from its period of infancy to the point of becoming ubiquitous in biomechanics. The development of increasingly complex and sophisticated FE models has contributed to a comprehensive understanding of the *in situ* mechanical response of biological systems (Yang et al., 2006).

In the present study, micro Computed Tomography (μCT) and FE modeling techniques were employed to determine whether different masticatory forces and movements are able to significantly alter the stress/strain equilibrium of the mouse mandible. The obtained results indicate that food consistency may be associated with micro evolutionary modifications in rodent mandible morphology that will overall impact on skull shape adaptations.

## Materials and methods

A mouse skull (C57Bl/6-Sv129 genetic background) was scanned by μCT in order to reconstruct a 3D model required for the intended analysis. This technique is capable of producing 2D images of various structures, based on their ability to withstand the emitted X-radiation. As bone and all other hard tissues (e.g., enamel, dentine) have a unique spectrum of X-ray permeability, they shade in different tones of white/gray within a CT slice, thus allowing their relatively unhindered segmentation with some minor overlapping of hard tissue types. This resulted in a 2D outline of the various model entities within every scan, while the 3D geometrical data set was generated by overlaying consecutive slices. Data acquisition was in accordance to DICOM (Digital Imaging and Communications in Medicine).

Most of the popular segmentation methods are based on purely automated methodologies reconstructing ^*^.stl files (triangle surface models) of the bone contour through software programs. There exists, however, a consensus throughout literature that highly accurate models (on the micro scale) require semi-automated segmentation, supported by manual correction of the threshold results by experienced operators. A multi threshold segmentation technique was employed for the purpose of this study and the mean gray-scale within the image was calculated by employing sensitive edge detection filters, to distinguish the apparent tissue types (Rathnayaka et al., 2010). A bio realistic representation of all model entities was achieved through sequential segmentation of specific gray values (e.g., bone and dentine). All model entities representing hard tissue were reverse engineered in independent steps and then combined into the final model. This was favorable in terms of quality of the generated surfaces, as the overlapping density spectrums would otherwise result in loss of surface quality due to extensive noise in either one of these model entities. Missing model entities (i.e., soft tissue) were manually re-constructed (in ANSA 15.2 by Beta CAE Systems S.A.). The correction of the defects required further attention in order to develop a model capable of accurately representing all details (external and internal) vital to the analysis.

As the project focused on the mouse mandible, only this part of the geometry was considered during the analysis to increase computational efficiency and results accuracy. Once the 3D model was reconstructed, the file was imported in a mesh-oriented pre-processor and translated into a volumetric model.

The development of a mesh independent grid was considered an intrinsic aspect of developed model, as achieving a sufficient degree of bio-realisticity is a problem inherent to computational biomechanics (Tsouknidas et al., 2013). In this sense, the verification of the theoretical model was achieved through entity based convergence studies (Zienkiewicz and Taylor, 1989). This ensured the use of optimum mesh density in terms of processing time vs. results accuracy, while maintaining vital geometric characteristics (feature lines) throughout the model.

Two masticatory scenarios were identified, gnawing (incisal biting), and chewing (molar biting) and both of them examined for two load intensities, corresponding to a food type each (i.e., soft and hard food pellets). Gnawing was simulated with a purely vertical load applied at the tip of the incisors whereas chewing considered a loading direction inclined by 30° to the dorsal-ventral axis of the molars. The fracture strength of these pellet fragments was determined experimentally, based on uniaxial compression tests, and applied as the masticatory load during the two biting scenarios.

As the size of the food pellets was disproportionally large to the mouse skull dimensions, the gnawing/chewing load was established on the basis of fragmented pellet bites fitting the animal's mandible size. The fragments, considered during the compression tests, were in these terms similar in size to the rodent's oral cavity e.g., slightly wider than both incisal edges, while height and depth were restricted to the inter-incisor distance. These loads were equally distributed over the molars and incisors (both sides) as literature advocates bilateral biting to be more realistic than unilateral (Weijs and Dantuma, 1975).

The temporomandibular joint was simulated by articulating the mandible surface contacting the temporomandibular disc at its medial-lateral axis. Muscle forces were applied at their anatomical attachment points, according to previously reported results on the masticatory muscle architecture and its involvement in the initiation and stabilization of the mandible movement (Hautier and Saksiri, 2009). The masticatory musculature was considered to produce a combined force inversely proportional to the fracture strength of the food type (applied for each biting scenario on incisors or molars). These loads were allocated among the main six muscles, which are involved in rodent mastication (Cox et al., 2012), as follows:

➢ The internal pterygoid (a muscly highly active during the incisal power stroke) was considered to bare 23% of the superoinferior load (F_z_), while not contributing at all to the anteroposterior stabilization of the mandible. Its fibers insert on the medial fossa and the dorsal surface of the angular process and its attachment surface was simulated by a set of 2016 element nodes.
➢ The medial masseter pars zygomaticomandibularis is a major contributor to proximal movements of the mandible and was thus loaded with 24% of the mandible closing force (F_z_) and 30% of the anterior-posterior stabilization force (F_x_). It was simulated as being attached on a set of 953 nodes in the area of the masseteric fossa and the masseteric crest ventral to the first and second molars.
➢ The medial masseter pars zygomaticomandibularis was inserted on the ventral margin of the angular process of the mandible (covering a set of 3,242 superficial masseter pars reflexa elements). It was considered as the strongest among all masticatory muscles contributing 36% of the vertical (F_z_) and 30% of the ventral-dorsal load (F_x_).
➢ The posterior fibers of the temporal muscle, only involved in mandible retraction, were not considered during the biting scenarios. The posterior part of the temporal muscle attaches on the anterior border of the ramus of the mandible initiating from the short coronoid process up to the last molar covering a total of 782 element nodes. Due to its small proportions, the temporal muscle was considered to bare only 5% of the mandible closing force (F_z_) providing however 40% of the anterior-posterior stabilization (F_x_).
➢ The external pterygoid was completely neglected during the simulation, as it mainly provides stability between the condyle of the mandible and the temporomandibular joint disc. The external pterygoid contributes predominantly to movements such as mouth opening and retrusion/ipsilateral jaw movement but has a rather insignificant role during gnawing or chewing.
➢ The medial masseter posterior was also ignored during the simulation as it is significantly reduced when compared to the anterior part of the muscle, thus having a rather insignificant role in the production of masticatory loads.

Following this, the load transfer throughout the mandible was determined by a linear elastic analysis of the model in Abaqus 6.2 (by Simulia). The material properties applied to each tissue type are summarized in Table 1.

**Table 1:** **Material properties of the rodent's mandible**.

	**E [GPa]**	**Poisson ratio**
Cortical bone (Williams and Edmundson, 1984)	19.92	0.3
Alveolar Bone (Yu et al., 2010)	0.345	0.38
Enamel (Yu et al., 2010)	84.1	0.33
Dentin (Yu et al., 2010)	18.6	0.31
Pulp (Yu et al., 2010)	0.002	0.45
Mesenchyme (Rees and Jacobsen, 1997; Trickey et al., 2006)	0.1	0.37
Periodontal ligament (Rees, 2001)	0.05	0.45

## Results

**Figure 1:**
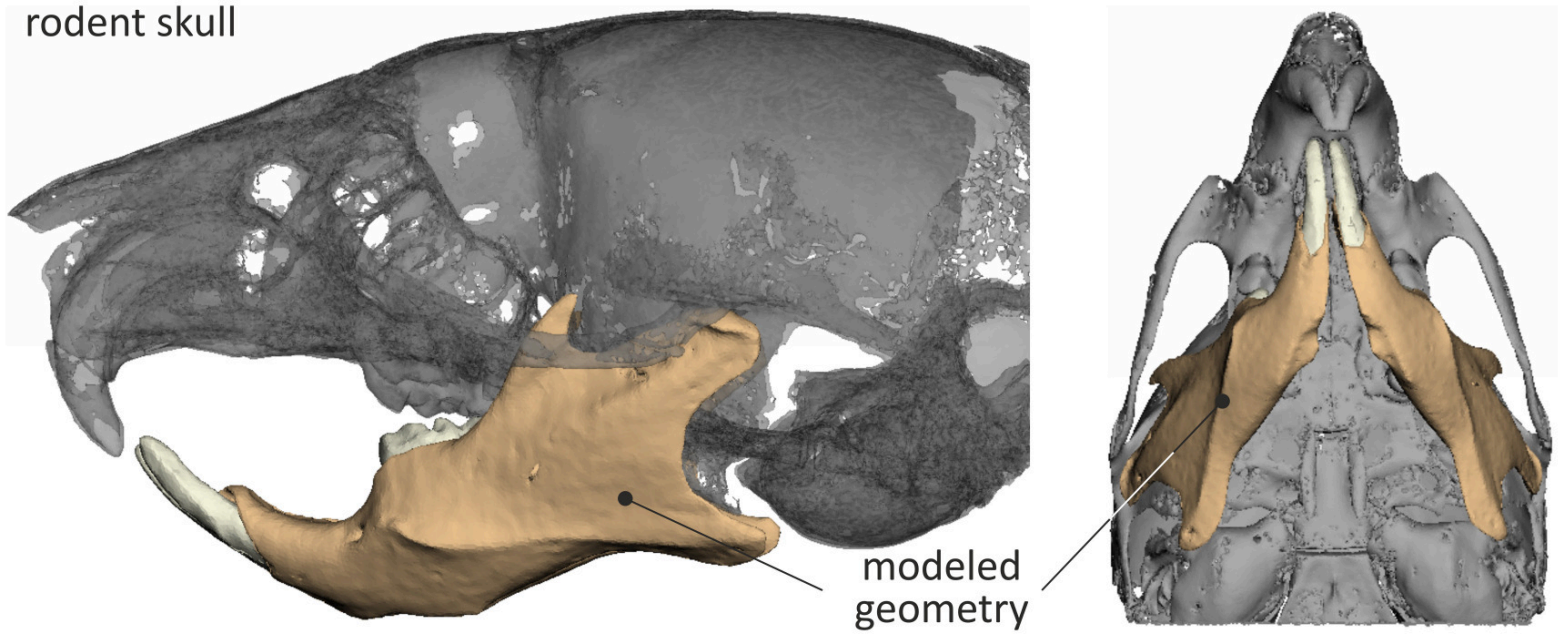
**3D skull model of the mouse and mandible considered during the analysis**.

The final STL model of the mouse skull, reverse engineered through μCT is shown in Figure 1.

The forces resulting for each one of the scenarios are summarized in Table 2. The mastication loads were equally distributed over the molars and incisors (both sides).

**Table 2:** **Loads applied for each biting scenario**.

**Load [N]**	**Gnawing (incisors)**		**Chewing (molars)**	
	**Soft pellet**	**Hard pellet**	**Soft pellet**	**Hard pellet**
Superoinferior [F_z_]	0.84	2.5	0.42	1.26
Anteroposterior [F_x_]	0	0	0.42	1.26

**Figure 2:**
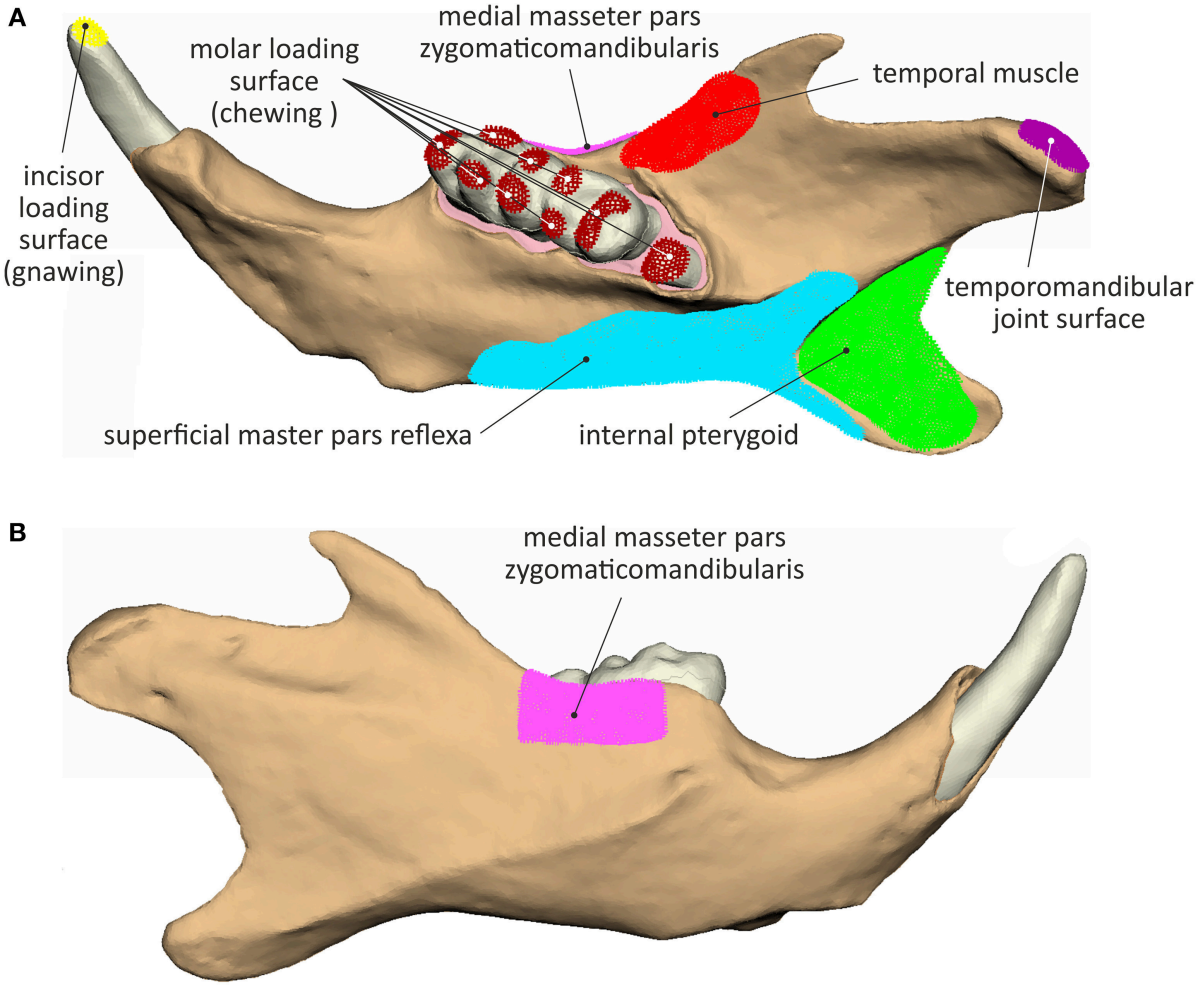
**Muscle attachment, load application and joint surfaces considered during the analyses: (A)** lingual view and **(B)** buccal view.

The temporomandibular joint was simulated by articulating the mandible surface contacting the temporomandibular disc at its mediolateral axis (Figure 2). An overview of the muscle attachment and loading surfaces, considered during simulation is provided in Figure 2.

### Loading scenarios

The different loading scenarios resulted in significant variations of the stress and strain patterns developing on the mouse teeth and mandible. Biting types (i.e., gnawing or chewing) had a dominant effect on the stress fields experienced by the mandible, with loading intensity resulting in an almost linear stress increase.

### Mandible loading

**Figure 3:**
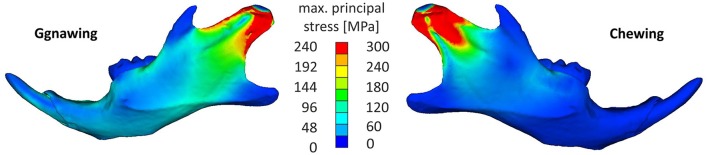
**Characteristic stress field developing in the mandible for a gnawing and a chewing scenario (hard pellets)**.

The analysis indicated that the masseter ridge was one of the most stressed areas of the mandible, with incisal biting (gnawing) also resulting in stress augmentations of the mental foramen of the mandible as well as on the temporomandibular joint (Figure 3). This can be explained with simple lever mechanics, since the loading point in incisal biting is more distant to the fulcrum (temporomandibular joint) than during molar biting (chewing). However, chewing resulted in stress concentrations that were tightly clustered in the in the dorsal part of the ramus, stressing the mandible to a higher intensity than the one observed during gnawing. Chewing resulted in higher stress values covering the area of the anterior margin of condylar articular surface to posterior-ventral tip of condyle up to the minimum of depression formed by condyle and processus angularis. This can be attributed to the tightly clustered musculature of the mandible, as the ventral-dorsal action of the medial masseter pars zygomaticomandibularis and the medial masseter pars zygomaticomandibularis counterbalance the bending moment during chewing, thus providing support to the articulated surface of the joint.

### Teeth loading

**Figure 4:**
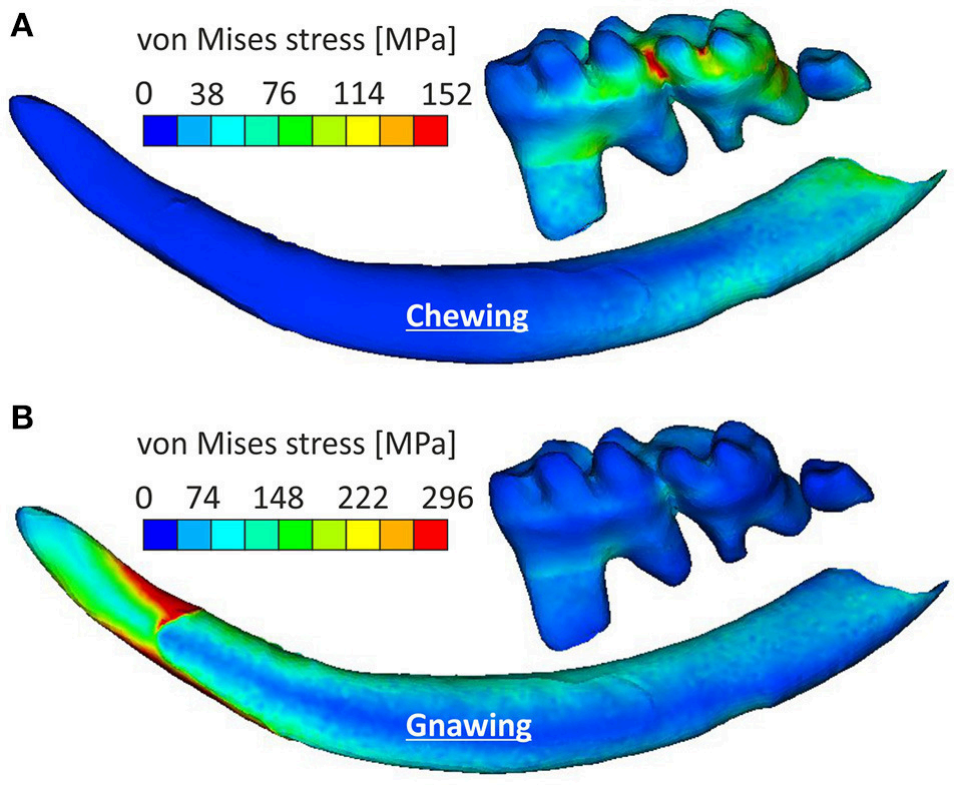
**Stress variations experienced by the incisor and molars for (A)** the chewing scenario and **(B)** the gnawing scenario.

Loading patterns occurring during chewing were more pronounced in molars when compared to incisors, where the stress was observed at their posterior part (Figure 4A). Chewing also provoked a slight anterior-posterior swift of the observed stress concentrations when compared to gnawing, which resulted in significant stress concentrations at the mandibular symphysis (Figure 3). During gnawing, the mandible engages an oscillatory movement, which introduces a ventral-dorsal component to both the force applied to the incisor and the counter-acting musculature. The distant nature of this load (applied on the incisor) to the temporomandibular joint contributes to a bending of the mandible that significantly increases the overall stress values during gnawing, which is especially noteworthy in the cross-section of the incisor spanning from the anterior margin of muscle insertion area on ventral site of incisor ramus to the minimum of depression on dorsal side of incisor ramus (Figure 4B).

### Periodontal loading

**Figure 5:**
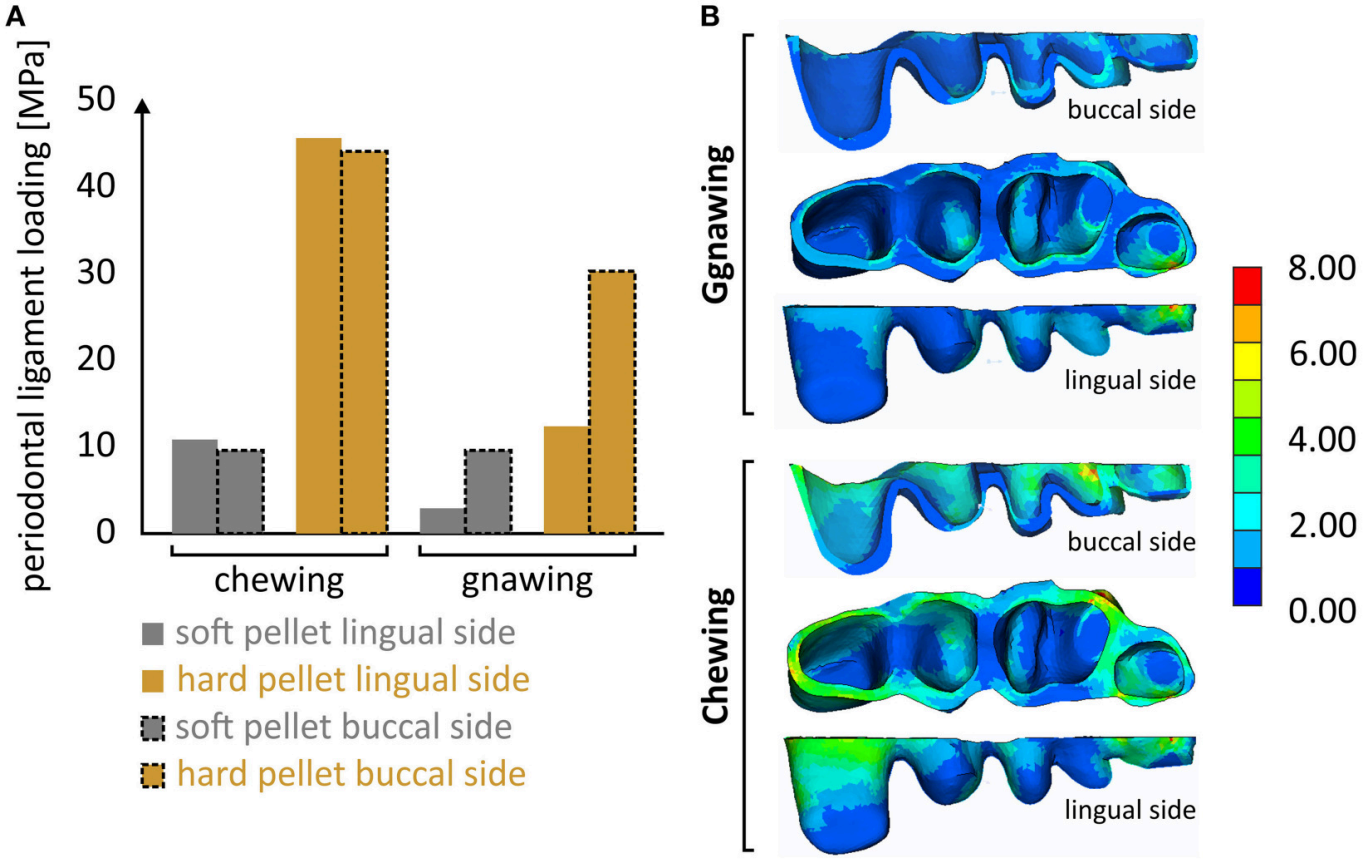
**(A)** Overview of the max. developing von Mises stress calculated at the periodontal ligament during the four loading scenarios and **(B)** characteristic developing stress fields during gnawing and chewing.

It is notable that the mastication forces during chewing were considerably cushioned by the periodontal ligament, thus preventing overloading of the alveolar bone and isolating the masseter ridge from abrupt loading. Although the results indicated a linear correlation of this effect to the applied load, this cushioning is in reality expected to fade along with an increasing masticatory force. This limitation is attributed to the linear-elastic nature of the introduced model, as there is no literature available concerning the nonlinear-viscoelastic material properties of the periodontal ligament. Different stress fields within the periodontal ligament were experienced during the various biting scenarios (Figure 5). It seems that the effect of biting type, although apparent, is less pronounced than that of the food type. Another noteworthy aspect is that incisal biting also resulted in stress accumulation in the periodontal ligament, even though no loads were applied at the molars. This is indicative of the overall deformation of the mandible during gnawing, which is significantly higher than the one observed during chewing, thus provoking strain induced stress throughout the mandible.

## Discussion

Despite the use of dentition and skull morphology to classify both living and extinct species (Machado-Allison and Garcia, 1986; Jégu, 2003), the etiology for this diversification remains unexplored. Even though recent studies have indicated bite forces as a key diversifying aspect in jaw evolution of gnathostomes (Grubich et al., 2012), no attempt has been made so far to separate these environmental effects from genetic ones.

This is the first attempt to associate the occurring masticatory forces to evolutionary aspects of rodent mandibles. The masticatory loads, adopted from the pellet compression tests, exhibited significant variations in magnitude and direction (see Table 2), while for each one of the scenarios correlated well to literature data (Cogelia et al., 1976; Freeman and Cliff, 2008). The choice of a laboratory mouse was based on the consensus that they are ideal model organism for developmental studies, providing two dental model systems, incisor and molars (Mitsiadis and Graf, 2009). Rodent molars resemble, but are not identical, to the human ones. In contrast, the rodent incisors have a cylindrical shape and differ from other teeth in that they are continuously growing organs that erupt throughout the life of the animal. All teeth interact actively with the alveolar bone through the periodontal ligament (PDL), a fibrous connective tissue structure, comprising of several cell types e.g., fibroblasts, progenitor cells etc. The mechanical stress, developing on teeth during mastication, is supported and modulated by this highly specialized tissue occupying the space between the tooth root and the alveolar bone. Despite its physiological importance (Rees, 2001), there is no literature available concerning the biomechanical response of this nonlinear and viscoelastic material. Determining the *in-situ* occurring stress fields in the periodontal ligament is vital in assessing its cellular response, as the values provided in Figure 5 can be applied *in-vitro*, to experimentally assess the mechanobiology of the cells endemic to this tissue.

Rodents exhibit two mutually exclusive biting modes (Cox et al., 2012): incisal biting (also called gnawing) and chewing at the molars. Due to a misaligned mandible/cranium molars and incisors cannot be in occlusion simultaneously, forcing the mandible into a propalinal movement to activate both biting modes (Becht, 1953; Hiiemae and Ardran, 1968).

Recent studies observed phenotypic differentiations in laboratory bread mice and associated them to food consistency and muscle driven remodeling (Renaud et al., 2010). This coincides with the results presented here, as biting type had a dominant impact on the loading patterns experienced by the mandible. This suggests that mice subjected to feeding ecologies biased toward gnawing should experience different postnatal growth patterns of the mandible than those biased toward chewing, thus hinting toward the etiology of recent findings.

It remains however unclear whether the trigger for this plastic response is due to environmental influences or genetic effects, as it stands to reason that the recorded stress variations will also affect cell proliferation or differentiation events thus influencing additional bone or other tissue formation based on biting type and intensity (Haudenschild et al., 2009). Gnawing induces a significantly higher bending moment on the mandible than chewing, a process that is largely compensated by the active musculature, which counter intuitively reduces the stress fields developing on the ascending ramus. As a result chewing only provokes stress concentrations in the alveolar region of the mandible whereas the distal nature of the load applied on the molars during chewing is absorbed mainly by the mandibular bone invoking stress concentrations at the masseter fossa.

The results shown in this study attest that gnawing can double the stress intensity experienced by areas of the mouse mandible when compared to chewing, while also increasing the strain of both, bony and dental tissue. A response that is in agreement with recent literature findings, which indicated that gnawing induced the highest mean stress across the skull (Cox et al., 2013). Previous studies in other species have suggested that a functional link also exists between tooth morphology and feeding ecology (Wainwright et al., 2004).

Since every form of life, from cells to organisms is mechanosensitive, mechanical stimulus is widely accepted to regulate the growth and development of any tissue type under physiological conditions (Farng et al., 2008). While cell responses to physical forces have been exhaustively studied *in vitro* (Orr et al., 2006; Gurkan and Akkus, 2008), there is an evident lack of literature concerning the effect of extracellular forces on cell behavior *in vivo* (Delaine-Smith and Reilly, 2011).

Future work includes the use of the presented Finite Element model, in combination with *in-vitro* experiments, to determine the *in-situ* response of cells to masticatory forces. Cells extracted from the periodontal ligament will, in these terms, be subjected to the calculated stress values (presented in Figure 5). This is expected to provide refined insight to masticatory-induced migration and proliferation of cells endemic to the periodontal ligament.

## Author contributions

AT and TM: Contributed to the conception of the hypothesis of the study, collaborated in the development of the model and was involved in the evaluation of the results and preparation of the manuscript. He also provided approval for the publication of this version. LJ: Contributed to the development of the model and the interpretation of data for the work. She was also involved in the preparation of the manuscript and provided approval for the publication of this version. EK: Contributed to the development of the model, the acquisition and the analysis of data for the work. He was also involved in the preparation of the manuscript and provided approval for the publication of this version. NM: Contributed to the development of the model, the analysis and the interpretation of data for the work. He was also involved in the preparation of the manuscript and sanctioned the publication of this version.

### Conflict of interest statement

EK was employed by company BETA CAE Systems S.A. The other authors declare that the research was conducted in the absence of any commercial or financial relationships that could be construed as a potential conflict of interest. The handling Editor declared a past co-authorship with two of the authors TM and LJ, and states that the process nevertheless met the standards of a fair and objective review.
